# Chitosan Films in Food Applications. Tuning Film Properties by Changing Acidic Dissolution Conditions

**DOI:** 10.3390/polym13010001

**Published:** 2020-12-22

**Authors:** Elodie Melro, Filipe E. Antunes, Gabriela J. da Silva, Inês Cruz, Philippe E. Ramos, Fátima Carvalho, Luís Alves

**Affiliations:** 1 CQC, Department of Chemistry, University of Coimbra, 3004-535 Coimbra, Portugal; elodie.melro@uc.pt (E.M.); fcea@ci.uc.pt (F.E.A.); 2 Center for Neurosciences and Cell Biology and Faculty of Pharmacy, Health Sciences Campus, University of Coimbra, 3000-548 Coimbra, Portugal; gjsilva@ci.uc.pt; 3 Primor Charcutaria—Prima S.A., Avenida Santiago de Gavião, 1142, 4760-003 Vila Nova de Famalicão, Portugal; ines.cruz@primor.pt (I.C.); philippe.ramos@primor.pt (P.E.R.); fatima.carvalho@primor.pt (F.C.); 4 CIEPQPF, Department of Chemical Engineering, University of Coimbra, Rua Sílvio Lima, Pólo II, 3030-790 Coimbra, Portugal

**Keywords:** acetic acid, chitosan, citric acid, film, food preservation, lactic acid

## Abstract

Food contamination due to the presence of microorganisms is a serious problem. New food preservation systems are being studied to kill or inhibit spoilage and pathogenic microorganisms that contaminate food and reduce the shelf life of products. Chitosan films with potential application to food preservation have witnessed great developments during the last years. Chitosan is a cationic polysaccharide with the ability to form films and possess antimicrobial properties. It is water-insoluble but can be dissolved in acidic solutions. In the present work, three different acids (acetic, lactic and citric) were used in chitosan dissolution and both, the resultant solutions and formed films were characterized. It was observed that chitosan water-acetic acid systems show the highest antimicrobial activity due to the highest chitosan charge density, compared to the mixtures with lactic and citric acid. This system showed also the higher solution viscosity compared to the other systems. Chitosan–acetic acid films were also the ones presenting better mechanical properties; this can be attributed to the fact that lactic and citric acids remain in the films, changing their properties, which does not happen with acetic acid. Films produced from chitosan dissolved in water/acetic acid system are resistant, while very fragile but elastic films are formed when lactic acid is used. It was demonstrated that a good selection of the type of acid not only facilitates the dissolution of chitosan but also plays a key role in the properties of the formed solutions and films.

## 1. Introduction

Microbial degradation and dehydration of meat products during their shelf life were identified to be the parameters that present a higher influence in meat products quality and consumer acceptance [1]. The properties of the products are affected by the storage conditions, i.e., storage temperature, but also by the preservatives used to control microorganism’s proliferation. Most of the preservatives used nowadays are synthetic compounds, such as sodium and potassium nitrite, sodium and potassium nitrate. The development of alternatives to those synthetic preservatives, preferentially bio-based options, able to keep the microorganisms counts in acceptable values and with no negative effects on human health, is a topic of major interest in food science.

Chitosan (poly β-(1→4)*N*-acetyl-d-glucosamine) is the deacetylated form of chitin [2] and during long time was considered a waste product of the crabbing and shrimp canning industry [3]. This linear polysaccharide has attracted considerable interest because is a non-toxic, biodegradable, biofunctional and biocompatible polymer [4,5] and has antimicrobial, antifungal, antitumor and hypocholesterolemic activities [6,7].

Chitosan also presents very good film-forming capacity [8], being highlighted its use in building edible films and coatings with antimicrobial properties to improve food safety and shelf life [4,9]. The antimicrobial activity of chitosan was already demonstrated, being proposed three different action mechanisms: (i) cell leakage owing to electrostatic interaction between positively charged chitosan and negatively charged microbial cell membranes; (ii) the penetration of chitosan into the nuclei of the microorganisms inducing the inhibition of the mRNA and protein synthesis; and (iii) the formation of an external barrier, chelating metals and causing the suppression of essential nutrients to microbial growth [10].

Chitosan is a weak base and it is insoluble in pure water and organic solvents [11], but can be solubilized in diluted acidic aqueous solutions, below pH 6.0. This characteristic is due to the presence of amino groups, whose pKa value is ca. 6.5 [12], and thus the insoluble-soluble transition occurs between pH 6.5 and 6.0. Below this value, the amines get protonated and chitosan becomes a soluble polymer, due to counterions entropy gain. On the other hand, above this range is insoluble owing to deprotonated amines [13]. Several organic and inorganic acids are reported to effectively dissolve chitosan, including acetic, formic, L-glutamic, lactic, hydrochloric and malic acid [14]. Organic acids are natural substances found in various fruits and fermented products that besides being effective to dissolve chitosan, some of them exhibit antimicrobial activity against foodborne pathogens [15].

In the present study, solutions/films of chitosan were prepared by dissolving the polymer in acetic, lactic and citric acid aqueous solutions and the properties of the obtained solutions/films were characterized. In literature is possible to find some studies dealing with chitosan dissolution using different acids [16,17], the influence of the acid type in the solution and film properties [18,19]; however, only a few studies address the effect of the acid used in chitosan dissolution in antimicrobial properties [20]. Here, we explore the effect of the three acids used in the dissolution of chitosan, and also the correlation of the used acid with the properties of the solution, in the films formed and their antimicrobial activity, discussing the possible mechanisms of bacterial inhibition and the effect of the residual acids in the formed films.

## 2. Materials and Methods

### 2.1. Materials

Chitosan, (extracted and purified from crab shell, deacetylation ≥ 85% and Mw of ca. 370 kDa) was purchased from Sigma-Aldrich (Merck), Algés, Portugal. Acetic acid and lactic acid were acquired from Panreac Química S.L.U., Barcelona, Spain, and citric acid from Merck, Algés, Portugal. For microbiological tests, Mueller Hinton broth was purchase from Sigma-Aldrich (Merck), Algés, Portugal. All chemicals were used as received without any further purification.

### 2.2. Methods

#### 2.2.1. Preparation of Chitosan Solutions and Films

Chitosan, medium molecular weight, was dissolved in acidic aqueous solutions, using acetic, lactic and citric acid at different concentrations, ranging from 2.0 to 10.0 wt. %, by stirring the mixtures until a homogeneous solution is obtained. After complete dissolution, chitosan solutions were centrifuged at 3600 rpm (ca. 2200× *g*) for 10 min to remove any residual undissolved polymer. Fifteen grams of each solution was then cast in a plastic Petri dish with a diameter of 90 mm and dried in the oven at 50 °C overnight.

#### 2.2.2. Characterization of Chitosan Solutions

The zeta potential was determined using a Malvern Zetasizer Nano ZS (Malvern Instruments, Malvern, UK) being the samples loaded in a folded capillary electrophoresis cell (Malvern Instruments, Malvern, UK); the Smoluchowski model was the model employed in the zeta potential determinations. The average values of zeta potential were calculated with the data obtained from three runs of triplicates, at 20 °C. The solution rheological properties were acquired using a Haake Mars III rheometer (Thermo Fisher Scientific, Schwerte, Germany), coupled with Peltier temperature control and set with a geometry cone-plate (C35/1°). Rotational tests were applied to the different samples and the Newtonian shear viscosity value, i.e., the plateau at low stresses of the viscosity profile, was extracted using Rheowin 4 Data Manager software and the appropriate model. The samples were measured at 5 and 20 °C.

Evaluation of the antibacterial activity was performed by determining the minimum inhibitory concentration (MIC) by using the broth micro-dilution method, according to the Clinical and Laboratory Standards Institute (CLSI, 2018) guidelines [21]. MIC is defined as the lowest concentration (in mg·L^−1^) of an agent that inhibits the visible growth of bacteria after overnight incubation. Briefly, the procedure involves the use of 96-well microtitration plates, where a volume of Mueller-Hinton broth was placed in each well; the same volume of the sample agent was added in the first well and successive 1:2 dilutions were performed. As a representative of a Gram-negative and Gram-positive cell bacteria, the antibiogram quality control strains *Escherichia coli* ATCC 25,922 and *Staphylococcus aureus* ATTC 29,213 were used, respectively. Direct colony suspension was performed until the turbidity matched that of a 0.5 McFarland standard. The inoculum should have approximately 5 × 10^5^ CFU/mL. The results were interpreted after 18–24 h of incubation at 35 °C. Growth and purity controls were included. Assays were performed in triplicate in two independent experiments for each strain.

Statistical analysis was performed using one-way ANOVA (α = 0.05) to evaluate significant differences between the zeta potential of the solutions obtained with the use of different acids.

#### 2.2.3. Characterization of Chitosan Films

Infrared (IR) spectra were recorded with a spectrophotometer Thermo Nicolet, IR380 (Thermo Scientific, Waltham, WA, USA) equipped with Smart Orbit Diamond ATR system. Fourier transform infrared (FTIR) spectral analysis was performed within the wavenumber range of 400–4000 cm^−1^. A total of 68 scans were run to collect each spectrum at a resolution of 2 cm^−1^ in the absorbance mode.

The values of force at break were obtained using a Texture Analyser TA.XT plus (Stable Micro Systems, Surrey, UK) following the ASTM D882-12 standard. Tensile grips with 35 mm were used to hold the specimens, which consisted of 40 mm × 40 mm film portions. Up to six repetitions were made for each sample using a grip and a probe speed of 1.0 mm·s^−1^. The presented results are based on the mean value of 6 measurements.

The opacity of the formed films was determined by measuring the absorbance of the films at 450 nm and dividing the absorbance value by the film thickness. The absorbance was recorded in a UV-Vis spectrophotometer, Shimadzu UV-2450 (Shimadzu Corporation, Tokyo, Japan), and the thickness was measured with a digital caliper (Wurth Portugal, Sintra, Portugal). Eight measurements were made for each sample and the mean value as well the standard deviation was estimated.

The brightness of the films was measured at 85° using a micro-TRI-gloss (BYK Gardner, Silver Spring, CO, USA). Fifteen measurements were made for each sample and the average values as well the standard deviation were calculated.

The hydrophobicity of the different films was estimated by contact angle measurements using OCA-20 equipment (DataPhysics Instruments GmbH, Filderstadt, Germany). Ten measurements were made for each sample and the average values as well the standard deviations were calculated.

Thermograms of chitosan films were measured using a thermogravimetric analyzer, TG 209 F Tarsus (Netzsch Gerätebau GmbH, Selb, Germany). Samples with ca. 6–8 mg were weighed in alumina pans and were heated from 30 to 1000 °C at a heating rate of 10 °C min^−1^ under N_2_ atmosphere (flow rate of 20 mL·min^−1^).

One-way ANOVA (α = 0.05) was used to perform statistical analysis to evaluate significant differences in the mechanical properties, contact angle, brightness and opacity of the produced films.

## 3. Results

### 3.1. Chitosan Solutions

**Figure 1:**
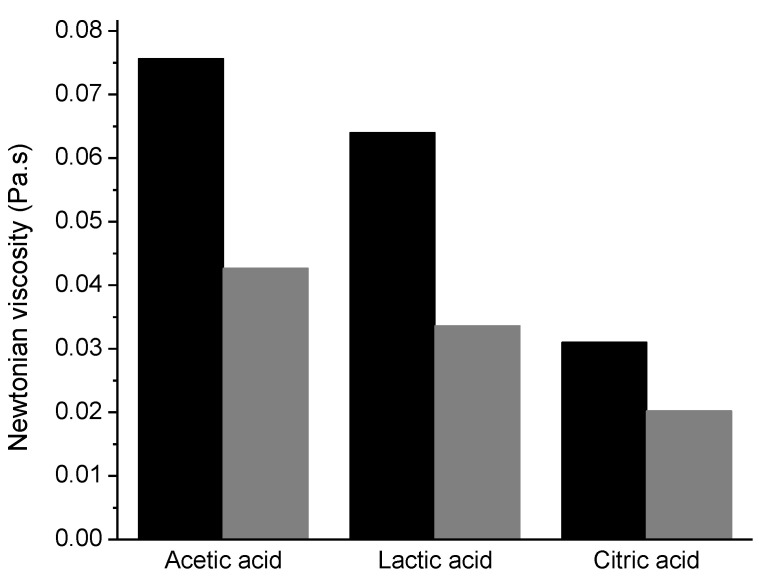
Newtonian viscosity of chitosan solutions (1.0% (*w*/*w*)) dissolved using different acids (2 wt. %), at 5 °C (black) and 20 °C (gray).

The acid choice plays a key role not only in chitosan dissolution but also in the properties of the solutions formed. One of the parameters affected is the viscosity of the solutions. In Figure 1, the Newtonian viscosity of chitosan solutions using acetic, lactic and citric acid is presented. Solutions with acetic acid show higher viscosities, while the viscosity decreases with the use of citric acid, due to the increase of shielding effect of the counter ion since these solutions had the highest ionic strength [22]. Furthermore, the temperature influences the viscosity of the solutions, being the solutions at 5 °C more viscous than at 20 °C; the solutions containing chitosan in citric acid are the least affected by temperature changes.

**Figure 2:**
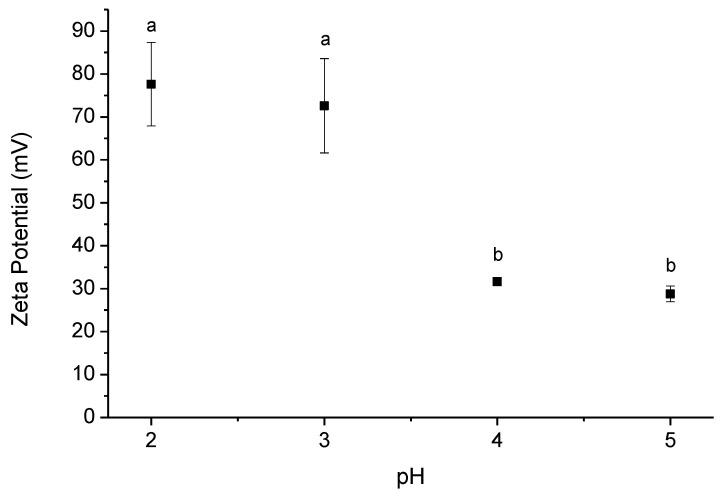
Zeta potential of chitosan solutions (0.5% (*w*/*w*)) dissolved using acetic acid at different pH’s. Data points with different letters are significantly different (*p* < 0.05) from each other. The error bars consist of the standard deviation calculated with the data obtained from three runs of triplicate.

**Figure 3:**
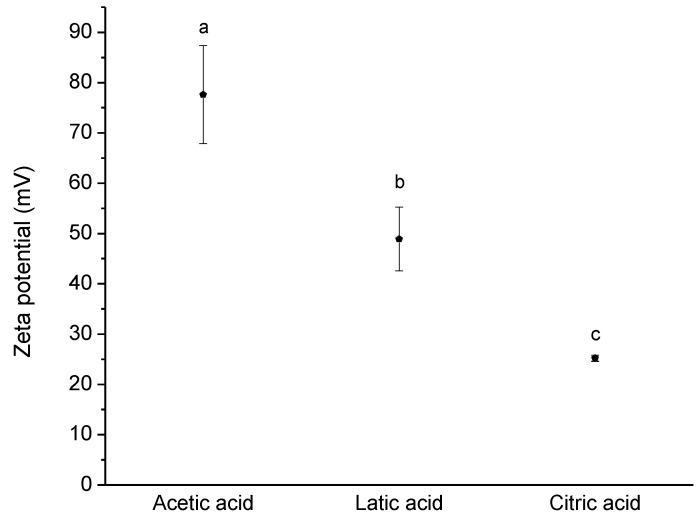
Zeta potential of chitosan solutions (0.5% (*w*/*w*)) dissolved with different acids at pH = 2. Data points with different letters are significantly different (*p* < 0.05) from each other. The error bars consist of the standard deviation calculated with the data obtained from three runs of triplicate.

Chitosan is a charged polymer at acidic pH values, being the zeta potential of its solutions extremely influenced by the pH of the solution and the acid used. The decrease of the pH value promotes the protonation of primary amines and consequently the increase of the charge density, as can be seen in Figure 2. Using acetic acid, the zeta potential is quite high, ca. +80 mV, in the pH range of 2–3. Furthermore, deep changes in the electrophoretic mobility were observed by changing the acid used, Figure 3.

To clarify the effect of the use of different acids in the preparation of the solutions on the charge density of the polymer, the zeta potential of chitosan solutions in acetic, lactic, and citric acid were measured. In Figure 3, it is notorious that the zeta potential is extremely influenced by the type of acid used for the dissolution of chitosan. The charge density using citric acid is quite low, being higher with lactic acid and even higher using acetic acid. Being expected an identical protonation of the amine groups to a specific pH value, independent of the acid used, the differences in the zeta potential can be explained by differences in the electrical double layer, according to the acid used. Looking to the partition coefficients of the used acids, it is possible to notice significant differences; Log P for acetic acid is −0.31 (at 20 °C) [23], −0.62 for lactic acid (20 °C) [24] and −1.65 (20 °C) for citric acid [25]. Thus, is clear that citric acid is the one that presents higher hydrophilicity compared to the other two acids. Ions like citrate are very water-soluble and a poor affinity with chitosan is expected; on the other hand, a better interaction is expected for acetate ions, due to their lower water solubility. The presence of acetate anions close to the chitosan surface can stabilize the electrical double layer, leading to a higher zeta potential value. On the other hand, the poor affinity of citrate results in a poor stabilization of the electrical double layer of chitosan, and consequently lower zeta potential is observed. The results obtained are in good agreement with other systems reported in the literature, and the effect of the acid ions is very well described for interactions of cations and anions with proteins [26]; as well, similar trends were reported for interactions of different acids with cellulose-based nanofibres [27]. Consequently, lower expansion of the chitosan chains is expected for the solutions using citric acid compared to the solutions containing acetic acid, leading to lower solution viscosity (Figure 1).

The sequence observed in zeta potential agrees with the sequence given above relating to the viscosity solutions. This means that the greater the charge density of the polymer is, the higher will be the viscosity solution, due to an increased polymer chain expansion and consequent better polymer chain entanglement.

The polycationic structure of chitosan is crucial to its antimicrobial activity [28]. The MICs of chitosan solutions containing acetic, lactic and citric acid against two structurally different bacteria, *E. coli* ATCC 25,922 and *S. aureus* ATTC 29,213, Gram-negative and Gram-positive bacteria, respectively, are showed in Table 1. There are no significant differences between the antibacterial activities against these two species, except for the acetic acid case. This observation can suggest a possible antibacterial mechanism: the positive charges of the amino groups of chitosan interact predominantly with anionic components (proteins, lipopolysaccharides (Gram-negative) and anionic lipoteichoic acids (Gram-positive) of the bacteria’s surface. These interactions cause a great change in the structure of the outer membrane, leading to the release of a major proportion of proteinaceous material from the cells [29,30]. This interaction of positive chitosan with the anionic components of the cell wall, destabilizing it, can prevent bacteria growth or even kill bacteria. However, an important difference in MIC is observed when using different acids, being the chitosan—acetic acid association that shows the better antibacterial activity. The used acids themselves also present some antibacterial activity (MIC of acetic acid 781 mg/L against *E. coli* and *S. aureus*; lactic acid presented a MIC of 1562 mg/L against *E. coli* and 1302 mg/L against *S. aureus*; citric acid showed a MIC of 2083 mg/L against *E. coli* and 651 mg/L against *S. aureus*). Comparing the results obtained by the isolated acids with the ones obtained with the interaction of chitosan with the different acids is obvious the great decrease observed for the combination of chitosan with acetic and lactic acids, contrary to the observed with citric acid, where an increase in the MIC was observed.

Previously we observed that the charge density of chitosan was influenced by the acid used; here we can confirm that the charge density is intimately related to the antimicrobial activity. Therefore, a better antimicrobial activity is reached by using acetic acid in chitosan dissolution, compared to the two other acids used.

### 3.2. Chitosan Films Characterization

**Figure 4:**
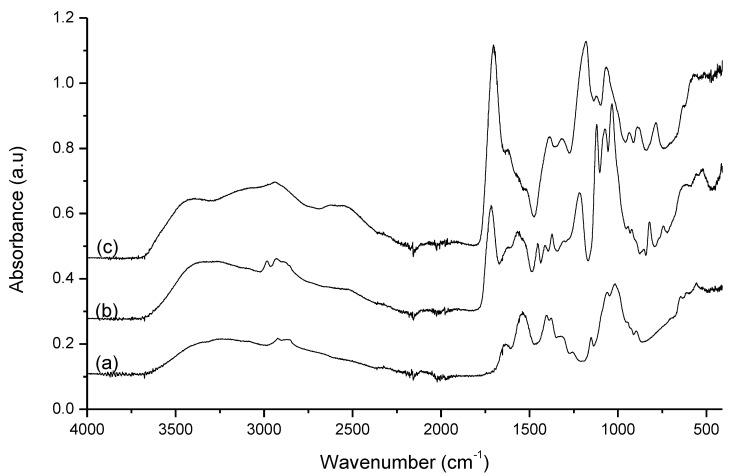
Normalized infrared spectra of chitosan films produced from a solution containing 1.5 wt. % of chitosan in solutions with 5 wt. % of (**a**) acetic acid, (**b**) lactic acid and (**c**) citric acid.

The IR analysis is an important tool to analyze the phase structure and the interaction between chitosan and the different acids. The spectrum of chitosan films with 5 wt. % of each acid is represented in Figure 4. The IR spectra of chitosan films using acetic acid present the same bands and similar intensities, independently of the amount of acetic acid added, meaning that there are no remarkable differences in the structure of these films. The skeletal vibration of C–O stretching appears at 1018 cm^−1^, the antisymmetric stretch C–O–C and C–N stretch at 1151 cm^−1^, the CH_3_ symmetrical deformation at 1377 cm^−1^ and the -CH_2_ bending at 1405 cm^−1^ [31]. The characteristic amine peak at 1590 cm^−1^ relative to NH bending [32] has shifted to 1540 cm^−1^, and the characteristic peak at 1650 cm^−1^, relative to C=O–NHR stretching [32], is shifted to 1635 cm^−1^, indicating that an interaction is occurring among the amine group of the chitosan and acetic acid [33]. Finally, the C–H stretch appeared at 2871 cm^−1^ and the O–H and N–H stretch is observed at 3270 cm^−1^ [31]. No characteristic bands of acetic acid are observed showing a good removal of this compound during the solvent casting at 50 °C.

In the case of chitosan films obtained from lactic acid solutions, are quite similar, differing only in the intensity of some bands with the increase of the concentration of lactic acid. The bands at 740, 821, 1122, 1216 and 1717 cm^−1^, are characteristics of lactic acid, which reveal the presence of lactic acid in the films due to the low volatility of this acid at 50 °C. It is also possible to observe characteristic bands of chitosan; the band relative to skeletal vibration of C–O stretching is in the range of 1030–1036 cm^−1^, presenting a small deviation in the presence of lactic acid. The bands at 1373 and 1412 cm^−1^, refer to CH_3_ symmetrical deformation and –CH_2_ bending, respectively [31], are also observed. As in the chitosan films from acetic acid solutions, the characteristic amine peaks (1590 and 1650 cm^−1^) were shifted to 1563 and 1625 cm^−1^, respectively. This means that also in these films an interaction between the amine of chitosan and the lactic acid is observed.

Contrary to the spectra previously described, the spectra of the films of chitosan produced from citric acid solutions present many differences, due to the presence of characteristic bands of citric acid, meaning that citric acid was not removed during film formation. Obviously, with the concentration used (5 wt. %), the characteristic bands of citric acid are highlighted. Due to the numerous absorption bands of the citric acid, the analysis of the spectra becomes complicated because occurs an extensive overlapping of the chitosan and the acid bands, and bands resulting from the probable interaction of chitosan with citric acid.

**Figure 5:**
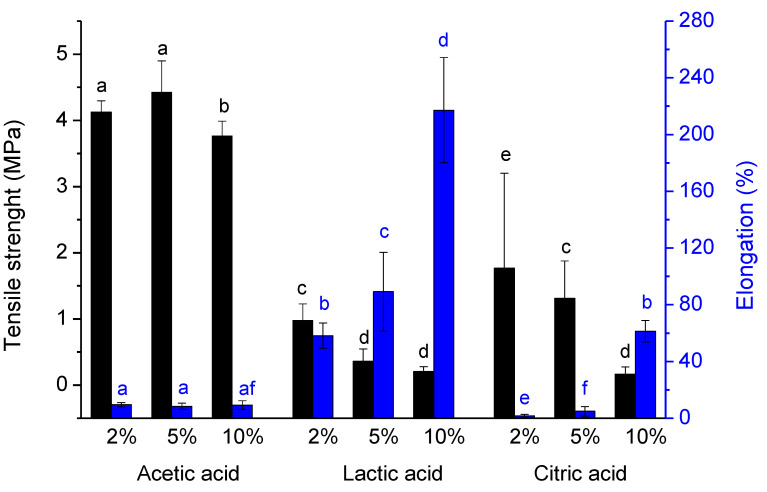
Tensile strength and elongation of chitosan films formed from 1.5% (*w*/*w*) chitosan solution containing different percentages of acetic, lactic, and citric acid. Data points with different letters are significantly different (*p* < 0.05) from each other. The error bars consist of the standard deviation obtained from six repetitions.

The tensile strength of the films was analyzed by measuring the force required to break the films. The results obtained, Figure 5, show that the choice of acid is crucial for the strength of the film. Films with acetic acid are the most resistant while films with lactic acid are the most fragile, due to the presence of lactic acid after film-forming, verified through the infrared analysis. Chitosan films with citric acid are greatly affected by the concentration of citric acid added, being the amount of acid inversely proportional to the strength of the film. The presence of high amounts of citric acid in chitosan films, concluded by FTIR analysis, increases the brittleness of the formed films. On the other hand, the higher tensile strength presented by the film formed from a chitosan solution in acetic acid can be attributed to two different factors: (1): the better dissolution of the biopolymer when acetic acid is used, as indicated by the rheological results, lead to a better entanglement of the polymer chains and consequently the network formed is stronger; also the higher zeta potential presented by solutions containing acetic acid leading to better polymer chain extension, and as consequence can interact better with neighbor chains reinforcing the polymer network. (2): the absence of residual acid allows the polymer chains to pack better and form stronger films.

The lower tensile strength obtained for the films formed from solutions of chitosan in lactic acid is a consequence of the residual acid on the film after film-forming, as previously alluded. Contrary to the films obtained from solutions of chitosan in acetic acid, the presence of lactic acid in the formed films resulted in poorer polymer chain packing, and thus weak but elastic films were formed. The poor packing and the consequent reduction in the interactions between the polymer chains, allow them to slide over each other, giving high elasticity to films.

Furthermore, brightness, opacity, and contact angle are parameters influenced by the type of acid used, as well as the percentage added, being these parameters shown in Table 2. In the case of the films formed with acetic acid, these parameters showed to be less influenced by the amount of acid used. On the other hand, when citric acid or lactic acid are used, the properties of the films presented changes. These changes are more notorious, especially in the parameters that are visually detected, like brightness and opacity, for the films produced from citric acid solutions; the brightness of the films decreases as the acid amount increases, being more pronounced the decrease between 2 and 5 wt. %, while the more opaque films are obtained from the solution with 5 wt. % of acid. All the films present hydrophilic character, as can be seen by the contact angle values, all lower than 90°, although the films formed with acetic acid have a slightly higher hydrophobicity. The lower contact angle (for the films obtained from lactic and citric acid) is probably related to the residual acid present in the films after film formation, due to the hydrophilic nature of these acids [24,25].

**Figure 6:**
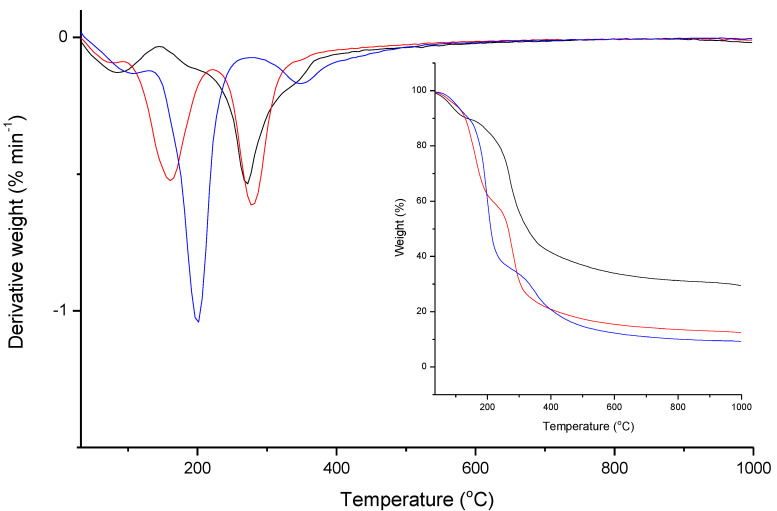
TGA thermograms and DTGA of chitosan films formed from chitosan solutions (1.5% wt. %) containing 5 wt. % of acetic acid (black line), lactic acid (red line) and citric acid (blue line).

The thermogravimetric analysis shows differences between the different films, Figure 6. Chitosan films prepared with acetic acid have two weight losses, being the first loss related to moisture evaporation and the second loss, initiated at 150 °C, related to chitosan decomposition [34]. In the case of films prepared with lactic and citric acid is verified the existence of three weight losses. Like films with acetic acid, there is a first step related to moisture evaporation and the last step relative to chitosan decomposition, started at higher temperatures, 200 and 260 °C for the film with lactic and citric acid, respectively. However, these films presented an intermediate step relative to the acid decomposition.

Similarly to FTIR, DTG analyses showed the presence of lactic and citric acid in the formed films, being the degradation of lactic acid at a lower temperature, ca. 160 °C and citric acid at ca. 200 °C. On the other hand, is possible to observe that the chitosan degradation happens at similar temperatures for the films prepared using acetic and lactic acid, in the range of ca. 280 °C and 290 °C, contrary to the degradation of chitosan in the films prepared using citric acid, which occurs at ca. 350 °C. This large difference in the degradation peak could be attributed to chitosan cross-linking by citric acid [35]. The cross-linking of the chitosan chains leads to a more rigid structure, which explains the delay in degradation temperature (Figure 6), and also the brittle films obtained with very reduced elasticity (Figure 5). The residual weight differences between the samples, which result from the carbonization during the degradation of chitosan above 350 °C, are related to the remaining acid quantity in the formed films; the residual amount, in the thermograms, for the film obtained from the solution acetic acid is higher than for the films containing lactic or citric acid, due to the absence of remaining acid in the film. Films produced from lactic or citric acid solutions behave like a blend, and as the carbonization temperature and the residual weight of these acids are lower than chitosan, the residual weight obtained for those films is lower. The TG results obtained in the present work are in agreement with other works reported in the literature for pure chitosan and chitosan blends, in terms of degradation profile and residual weight [36].

## 4. Conclusions

Chitosan solutions and formed film properties showed very distinct behavior when different acids were used. Solutions with acetic and lactic acid demonstrated good antimicrobial properties while the antimicrobial activity is much lower when citric acid is used. The use of citric acid also presented disadvantages in the formed films, since the acid is not easily removed during film formation, conditioning the properties of these films. One of the main differences observed is related to the tensile strength of the films prepared with acetic and lactic acid; films from solutions containing acetic acid were the strongest and lactic acid gives the less resistant films. The resistance of lactic acid to evaporation at 50 °C results in weak but elastic films; lactic acid acts as a plasticizer allowing the polymer chains to slide over each other. Therefore, depending on the functions of the chitosan film, it is important to select the acid and the appropriate amount, to have the appropriate properties to the film functionality.

In conclusion, we showed that the properties of a chitosan film are dependent on the acid type and its amount (concentration), and the interplay of these parameters is crucial for the function (or the use given) of the chitosan film.

## Figures and Tables

**Table 1:** Minimum inhibitory concentration (MIC) of chitosan dissolved using different acids against *Escherichia coli* ATCC 25,922 and *Staphylococcus aureus* ATTC 29,213.

	MIC (mg/L)	
	*E. coli*	*S. aureus*
Chitosan—Acetic acid (1:10)	90	150
Chitosan—Lactic acid (1:10)	160	160
Chitosan—Citric acid (1:10)	5730	4580

**Table 2:** Brightness, opacity and contact angle of chitosan films (formed from 1.5% (*w*/*w*) chitosan solution) with different percentages of acetic, lactic and citric acid.

Type of Acid	Acid Concentration (wt. %)	Brightness	Opacity (Abs_450nm_mm^−1^)	Contact Angle (°)
Acetic acid	2	30 ± 8 ^ab^	1.6 ± 0.2 ^a^	82 ± 4 ^a^
	5	35 ± 7 ^a^	1.2 ± 0.2 ^b^	84 ± 8 ^a^
	10	31 ± 4 ^ac^	1.4 ± 0.2 ^bc^	81 ± 7 ^a^
Lactic acid	2	28 ± 8 ^bc^	1.6 ± 0.3 ^ac^	74 ± 3 ^b^
	5	25. ± 9 ^b^	2.4 ± 0.4 ^d^	81 ± 4 ^a^
	10	20.0 ± 0.5 ^d^	2.5 ± 0.4 ^d^	73 ± 6 ^bc^
Citric acid	2	51 ± 8 ^e^	1.32 ± 0.06 ^b^	60 ± 10 ^d^
	5	15 ± 2 ^f^	12.8 ± 0.4 ^e^	70 ± 5 ^c^
	10	10 ± 3 ^g^	6.3 ± 0.3 ^f^	69 ± 10 ^b^

Same letters within a column indicate no significant differences (*p* > 0.05). The results presented consist of the average values and standard deviation of fifteen measurements.
